# Exploring the Hierarchical Influence of Cognitive Functions for Alzheimer Disease: The Framingham Heart Study

**DOI:** 10.2196/15376

**Published:** 2020-04-23

**Authors:** Huitong Ding, Ning An, Rhoda Au, Sherral Devine, Sanford H Auerbach, Joseph Massaro, Prajakta Joshi, Xue Liu, Yulin Liu, Elizabeth Mahon, Ting FA Ang, Honghuang Lin

**Affiliations:** 1 School of Computer Science and Information Engineering Hefei University of Technology Hefei China; 2 Key Laboratory of Knowledge Engineering with Big Data of Ministry of Education Hefei University of Technology Hefei China; 3 Department of Anatomy and Neurobiology Boston University School of Medicine Boston, MA United States; 4 Department of Epidemiology Boston University School of Public Health Boston, MA United States; 5 The Framingham Heart Study Framingham, MA United States; 6 Department of Neurology Boston University School of Medicine Boston, MA United States; 7 Department of Biostatistics Boston University School of Public Health Boston, MA United States; 8 Section of Computational Biomedicine Department of Medicine Boston University School of Medicine Boston, MA United States

**Keywords:** Alzheimer disease, neuropsychological test, stratification, Bayesian network, clustering

## Abstract

**Background:**

Although some neuropsychological (NP) tests are considered more central for the diagnosis of Alzheimer disease (AD), there is a lack of understanding about the interaction between different cognitive tests.

**Objective:**

This study aimed to demonstrate a global view of hierarchical probabilistic dependencies between NP tests and the likelihood of cognitive impairment to assist physicians in recognizing AD precursors.

**Methods:**

Our study included 2091 participants from the Framingham Heart Study. These participants had undergone a variety of NP tests, including Wechsler Memory Scale, Wechsler Adult Intelligence Scale, and Boston Naming Test. Heterogeneous cognitive Bayesian networks were developed to understand the relationship between NP tests and the cognitive status. The performance of probabilistic inference was evaluated by the 10-fold cross validation.

**Results:**

A total of 4512 NP tests were used to build the Bayesian network for the dementia diagnosis. The network demonstrated conditional dependency between different cognitive functions that precede the development of dementia. The prediction model reached an accuracy of 82.24%, with sensitivity of 63.98% and specificity of 92.74%. This probabilistic diagnostic system can also be applied to participants that exhibit more heterogeneous profiles or with missing responses for some NP tests.

**Conclusions:**

We developed a probabilistic dependency network for AD diagnosis from 11 NP tests. Our study revealed important psychological functional segregations and precursor evidence of AD development and heterogeneity.

## Introduction

### Background

Alzheimer disease (AD) is a chronic neurodegenerative disease characterized by cognitive decline [1]. Neuropsychological (NP) tests—a key measure of phenotypic expression of one’s cognition state—are commonly used by practitioners to assess cognitive dysfunction, especially in the memory, attention, and executive domains [2,3]. However, given the extensive variability in performance patterns across a standard comprehensive protocol of NP tests, physicians often find themselves making clinical decisions with certain degrees of uncertainty, and the situation is compounded when patients are unable to complete the tests because of a multitude of reasons. Given the data heterogeneity within and across NP tests, conventional qualitative classification is unable to accurately portray the clinical manifestation of a spectrum disorder such as AD. To date, many studies examined various cognitive domains individually [4], as separate entities, when in fact different regions of the brain work simultaneously and not in silos [5]. Therefore, to better characterize the complexity of AD, we need to identify and describe the hierarchical interaction pattern among NP tests and their symbiotic relationship with each other, to help illuminate the indices of neurodegenerative processes. Some researchers have proposed to focus on AD precursors of cognitive decline to reduce AD clinical trial failures [1]. We contend that the relationship is bidirectional. Different patterns of symptoms are indicative of cognitive impairment, whereas the presence of cognitive impairment impacts the symptoms associated with subsequent risk. Furthermore, to enhance clinical utility, a full global use of available observations will aid physicians with AD diagnosis, particularly for those patients who exhibit more heterogeneous NP profiles.

Many risk factors of AD have been identified in past decades [6,7]. Apolipoprotein E4 (ApoE4) status has been demonstrated to be a significant genetic risk factor for AD [8]. Although the factors underlying the sex differences have generally been weakly investigated, the difference indeed exhibits influence on the development and progression of AD [9]. ApoE4 tended to have different effects on AD between men and women [10]. In addition, education has been recognized as another potential risk factor, where people with different levels of education tended to show different risks of AD [11]. However, the hierarchical interplay between different risk factors and their effects on cognitive status are yet to be investigated.

### Objective

The objective of this study was to represent the intricate interplay of various NP tests with probabilistic graphical models and provide a top-down theoretical view to demonstrate the relationship between NP tests and cognitive status.

## Methods

### Study Population

The Framingham Heart Study (FHS) is a community-based longitudinal observational study that began in 1948. Details of FHS cohorts have been previously described [12]. Briefly, three generations of participants have been enrolled since 1948. To reflect the increasing ethnic diversity in Framingham, two additional cohorts, Omni Study 1 and Omni Study, were enrolled in 1994 and 2002, respectively. Every 2 to 8 years, each participant is given a comprehensive physical examination and queried for various lifestyles. NP tests have been administered through ancillary studies using standardized testing protocols and scoring procedures since 1981 [13]. Routine quality assurance processes were performed to keep consistency of these tests over time [14]. This study included all participants with valid NP tests from the original cohort (Gen I), offspring cohort (Gen II), multiethnic Omni 1 cohort, and new offspring spouse cohort [15]. Given the fact that AD primarily affects participants of advanced age, and the average age of dementia onset among FHS participants is around 85 years, our study was restricted to participants who were 70 years or older [16,17].

**Figure 1:**
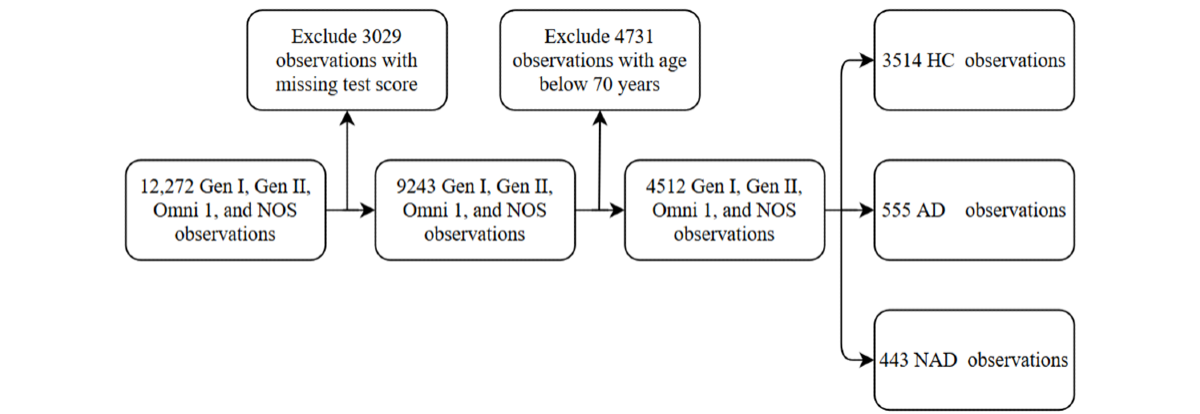
The process of sample selection. AD: Alzheimer disease; HC: healthy control; NAD: non-Alzheimer dementia; NOS: new offspring spouse.

The dementia diagnosis was based on the Diagnostic and Statistical Manual of Mental Disorders, fourth edition, whereas AD diagnosis was based on the National Institute of Neurological and Communicative Disorders and Stroke and the Alzheimer disease and Related Disorders Association [18]. All dementia diagnoses were adjudicated by an expert panel consisting of at least one neurologist and one neuropsychologist, using information from various sources such as NP assessments, neurology examinations, family interviews, FHS health exams, and external medical records [19]. According to their cognitive status, participants were grouped as healthy control (HC), AD, and non-Alzheimer dementia (NAD). Details of the dementia surveillance have been published [20-22]. The process of sample selection is shown in Figure 1.

### Identification of Cognitive Function Clusters

We performed correlation analysis to explore the dependency between NP tests. A cognitive function cluster is a set of NP tests that have stronger correlation than those outside of the cluster. The correlation between NP tests was assessed by Pearson chi-square test. Given the limited number of NP tests in our study, we used correlation coefficient 0.6 as the correlation cut-off, similar to previous studies [23,24]. Correlations between NP tests and cognitive status were evaluated by one-way analysis of variance [25].

### Bayesian Network for Modeling Hierarchical Probabilistic Dependencies

Bayesian network is a representative probabilistic machine learning method [26], which explores the information contained in experimental data to evaluate the probability of specific hypotheses. It can summarize a complex system into a simplified representation to capture the hierarchical interplay among components and provide insights on how each component influences others [27]. A Bayesian network is represented by a directed acyclic graph composed of nodes and edges. In this study, we used nodes to represent NP tests and cognitive status, and edges to represent the influence between nodes. For example, Test A–>Cognitive Status means that Test A is the parent node of Cognitive Status, and Cognitive Status is the child node of Test A. The edge direction suggests that Test A has an influence on Cognitive Status, which is formulated as the conditional probability of how the Cognitive Status depends on Test A. In contrast, given that Test A has no incoming edges, its probability does not depend on other factors. These dependency relationships could propagate through the network and influence downstream tests. It is worth to note that the conditional probability depends only on parent nodes but not grandparent nodes. For example, if we also observe Cognitive Status–>Test B, it means that Test B is only directly dependent on Cognitive Status but not grandparent node Test A, although Cognitive Status is dependent on Test A.

**Figure 2:**
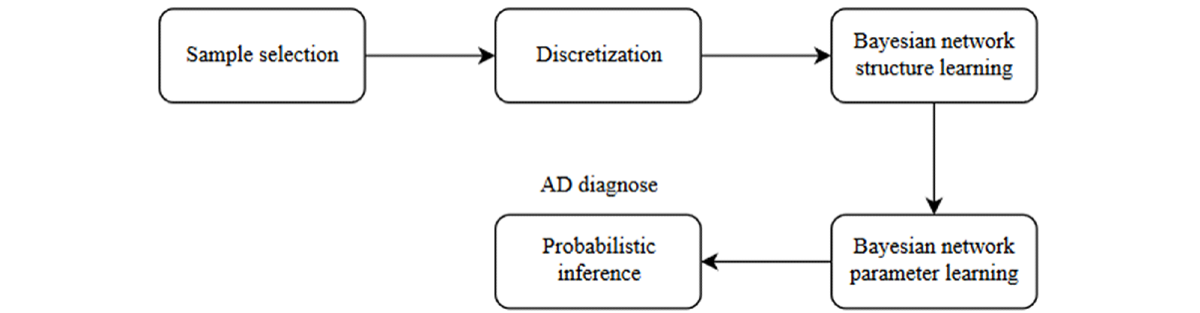
The flowchart of building a Bayesian network. AD: Alzheimer disease.

Figure 2 shows the flowchart of building a Bayesian network. Each observation includes 11 NP test scores and the cognitive status. Each continuous variable was discretized by partitioning around medoids method, which was used to find the intrinsic structures in NP tests and assign observations into homogeneous clusters [28,29]. The optimal number of clusters was determined by the silhouette width [30].

A search-and-score strategy was then used to build Bayesian networks from NP tests. The algorithm first assigned a likelihood score to each candidate structure. The score represented how well that structure fits the NP tests, which was evaluated by the Bayesian Information Criterion [31]. Unnecessary complex structures could fit existing data well but lack the generalizability to new data. Therefore, to recover the underlying Bayesian network structures, we included a penalty term equal to the Minimum Description Length score [32]. The method was previously shown to outperform other scoring functions such as Bayesian Dirichlet equivalence score, Akaike information criterion, and factorized normalized maximum likelihood [31,33]. Two searching methods were then used to find the optimal structure. One was Heuristic Hill-Climbing greedy search, which aims to optimize the local score [34] but cannot apply any prior knowledge about the expected structure of Bayesian network. The other one was Tabu search, which was used for validation and could search the space of directed graphs while escaping local optimum [35]. Bootstrap was adopted to minimize the uncertainty of the model [36].

In Bayesian network parameter learning, two parametric estimation methods were used, including maximum likelihood estimation and Bayesian parameter estimation [37]. To further validate the Bayesian network, logic sampling method [38] was used to generate simulated data based on learned Bayesian network and check whether it was consistent with prior information about the correlation of NP tests. Details of the learning process are provided in Multimedia Appendix 1, Methods.

### Markov Blanket to Select Neuropsychological Tests

Following the principle of the filter-based feature selection, an optimal subset of NP tests was derived from the data itself but not the performance matrix [39]. One key feature is conditional independence, which defines a sufficient subset S as follows [40]: S∈G is a sufficient subset of NP tests if and only if P(Status|G)=P(Status|S), where G is a set consisting of all 11 NP tests. Cognitive status is conditionally independent of other NP tests given S. The set of locally affecting variables is called the Markov blanket [41]. In Bayesian networks, the Markov blanket of cognitive status is a set of NP tests that consists of parent nodes, child nodes, and spouse nodes of cognitive status [41]. NP test A is the spouse of NP test B because they have common children. NP tests in Markov blanket are directly connected to cognitive status, which, therefore, determined the probability distribution of cognitive status. It provides a direction for detecting the potential causal cognitive functions for AD [42,43]. Pearson chi-square test was used to demonstrate the conditional independence between NP tests and cognitive status [44].

### Probabilistic Inference

Once Bayesian networks were built, each participant’s cognitive status was derived using an averaging likelihood weighting simulation method, which is an approximating inference method [34]. It calculated the posterior probabilities of cognitive status from observed NP tests. The details are provided in Multimedia Appendix 1, Methods.

To demonstrate heterogeneity of hierarchical influence of cognitive functions and cognitive status, the analysis was conducted for the full observations and also stratified by sex (male or female), ApoE4 status (OMIM 107741), and education level (beyond high school/high school graduate and below). Participants with missing education information were excluded from the education-stratified analyses (11 observations). Similarly, for the ApoE-stratified analyses, participants who did not consent to genetic analyses or without ApoE4 information were excluded (200 observations).

All participants had provided written informed consent. This study was approved by the Institutional Review Board of Boston University Medical Campus. The data collection was monitored by a National Heart, Lung, and Blood Institute Observational Study Monitoring Board and complied with the Strengthening the Reporting of Observational Studies in Epidemiology reporting guideline [45].

## Results

### Sample Characteristics

Our study included 4512 sets of NP tests from 2091 participants, primarily of European ancestry (1166 females, mean age 79 [SD 6] years). On average, each participant underwent 2.2 NP examinations. One examination of the participant is considered as a study sample. Table 1 shows the clinical characteristics of study samples.

Although 32 NP tests have been administered at the FHS (Multimedia Appendix 1), this study was focused on 11 NP tests that were administered to more than 85% of participants between 1999 and 2016. These tests included the first version of Wechsler Memory Scale Logical Memory Immediate Recall (LMi) [46], Logical Memory Delayed Recall (LMd) [46], and Logical Memory Recognition (LMr) [46]; Visual Reproductions Immediate Recall (VRi) [46], Visual Reproductions Delayed Recall (VRd) [46], and Visual Reproductions Recognition (VRr) [46]; and Paired Associate Learning Immediate Recall (PASi) [46], and the first version of Wechsler Adult Intelligence Scale similarities test (SIM) [47]. Given the importance in the measurement of confrontational word retrieval and verbal memory, our study also included Boston Naming Test 30 item Even Version (BNT30) and hard-pair scores from PASi and PASd [46,48].

**Table 1:** Clinical characteristics of study samples. A total of 4512 sets of neuropsychological tests from 2091 participants were included.

Characteristics			Healthy control (n=3514)		Alzheimer disease (n=555)		Non-Alzheimer dementia (n=443)	
**Age at** **neuropsychological** **exam (years)**								
	Mean (SD)			79 (6)		85 (6)		84 (6)
	Range			70-101		70-103		70-97
Male, n (%)			1521 (43.3)		179 (32.3)		220 (49.7)	
**Highest level of education attained^a^**								
	High school and below, n (%)^b^			1491 (42.5)		358 (65.2)		241 (54.5)
	Beyond high school, n (%)^b^			2019 (57.5)		191 (34.8)		201 (45.5)
**ApoE4^c^ allele**								
	ApoE4(−), n (%)^b^			2794 (82.9)		346 (65.3)		327 (79.2)
	ApoE4(+), n (%)^b^			575 (17.1)		184 (34.7)		86 (20.8)
**Neuropsychological** **test scores, mean (SD)**								
	**Verbal memory**							
		Logical Memory Immediate Recall		11.2 (3.7)		4.8 (3.8)		7.9 (3.9)
		Logical Memory Delayed Recall		10.2 (3.9)		3.0 (4.0)		6.5 (4.1)
		Logical Memory Recognition		9.4 (1.4)		7.1 (2.3)		8.5 (1.7)
	**Visual memory**							
		Visual Reproductions Immediate Recall		7.1 (3.0)		3.1 (2.3)		4.0 (2.5)
		Visual Reproductions Delayed Recall		6.1 (3.1)		1.6 (1.9)		2.7 (2.4)
		Visual Reproductions Recognition		2.6 (1.1)		1.3 (1.1)		1.7 (1.1)
	**New learning**							
		Paired Associate Learning Immediate Recall		12.8 (3.3)		8.4 (2.9)		9.9 (2.8)
		Hard score of Paired Associate Learning Delayed Recall		2.0 (1.3)		0.5 (0.9)		1.0 (1.1)
		Hard Score of Paired Associate Learning Immediate Recall		4.4 (3.0)		1.1 (1.7)		2.0 (2.0)
	**Abstract reasoning**							
		Similarities Test		15.5 (3.9)		9.8 (5.0)		11.6 (4.7)
	**Language and naming**							
		Boston Naming Test, 30-item Even Version		26.1 (3.4)		19.4 (5.9)		22.3 (5.4)

^a^Valid education data (n): Healthy control (3510); Alzheimer disease (549); Non-Alzheimer dementia (442).

^b^Values were calculated based on the subset with valid data.

^c^ApoE4: Apolipoprotein E4. Participants who did not consent to genetic analyses or with no ApoE4 information were excluded. Valid genetic data (n): Healthy control (3369); Alzheimer disease (530); Non-Alzheimer dementia (413).

### Correlation Clusters of Neuropsychological Tests

**Figure 3:**
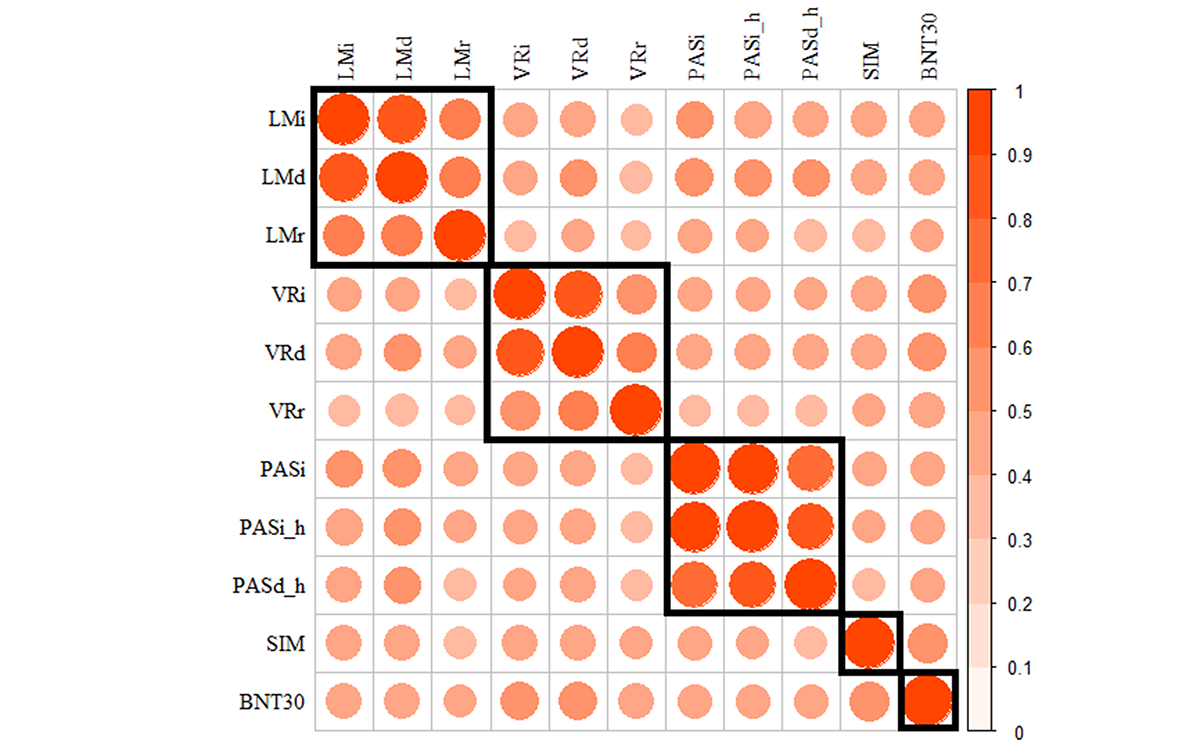
Correlation clusters between different neuropsychological tests. The red rectangles represent different clusters of tests. Bigger and redder nodes represent higher correlation, whereas whiter and smaller nodes represent lower correlation. BNT30: Boston Naming Test 30 item Even Version; LMd: Logical Memory Delayed Recall; LMi: Logical Memory Immediate Recall; LMr: Logical Memory Recognition; PASd_h: Hard score of Paired Associate Learning Delayed Recall; PASi: Paired Associate Learning Immediate Recall; PASi_h: Hard Score of Paired Associate Learning Immediate Recall; SIM: similarities test; VRd: Visual Reproductions Delayed Recall; VRi: Visual Reproductions Immediate Recall; VRr: Visual Reproductions Recognition.

We performed unsupervised clustering to investigate the correlation between NP tests. As shown in Figure 3, these NP tests could be divided into five clusters, each representing a distinct cognitive function. The intracluster correlation coefficients between NP test pairs were all higher than 0.60, which formed a clear cluster boundary to distinguish different cognitive functions without overlapping. In contrast, the intercluster correlation coefficients between NP test pairs were mostly lower than 0.50. The correlation of NP tests in subpopulations is shown in Multimedia Appendix 1.

As expected, three cognitive outcome groups had quite different mean NP test scores (Tukey-Kramer test, *P*<.001), suggesting a strong correlation between NP test and cognitive status (Multimedia Appendix 1). The association remained significant after Bonferroni correction for multiple testing.

### Bayesian Networks

**Figure 4:**
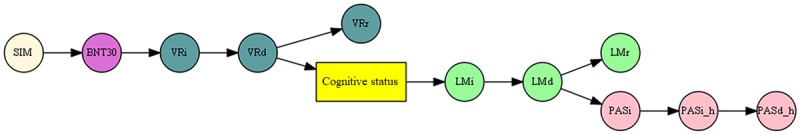
Bayesian network shows the hierarchical influence between neuropsychological tests and cognitive status. BNT30: Boston Naming Test 30 item Even Version; LMd: Logical Memory Delayed Recall; LMi: Logical Memory Immediate Recall; LMr: Logical Memory Recognition; PASd_h: Hard score of Paired Associate Learning Delayed Recall; PASi: Paired Associate Learning Immediate Recall; PASi_h: Hard Score of Paired Associate Learning Immediate Recall; SIM: similarities test; VRd: Visual Reproductions Delayed Recall; VRi: Visual Reproductions Immediate Recall; VRr: Visual Reproductions Recognition. Nodes with the same color represent NP tests measuring the same cognitive function.

**Figure 5:**
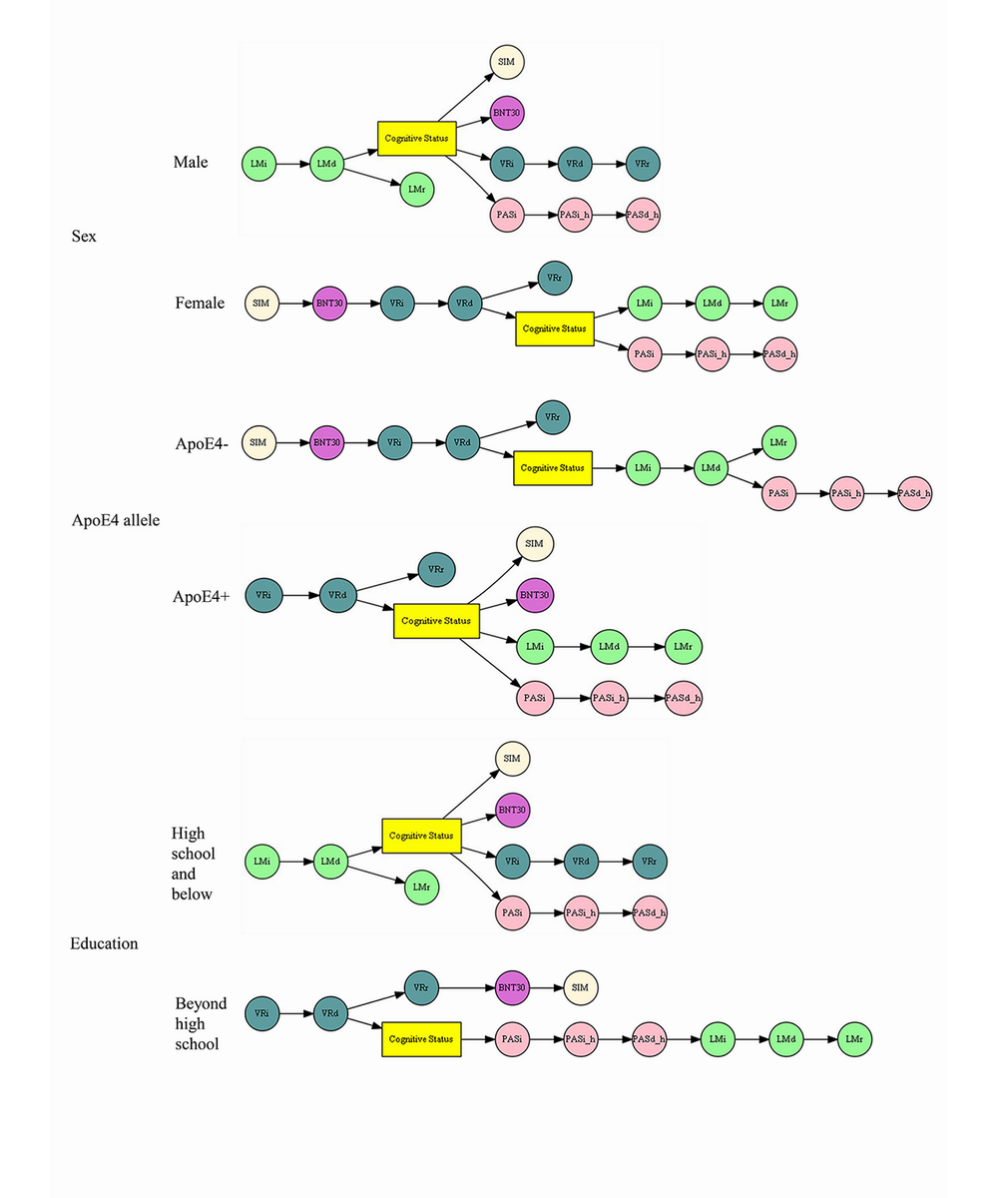
Hierarchical influence of neuropsychological tests and cognitive status in subpopulations. BNT30: Boston Naming Test 30 item Even Version; LMd: Logical Memory Delayed Recall; LMi: Logical Memory Immediate Recall; LMr: Logical Memory Recognition; PASd_h: Hard score of Paired Associate Learning Delayed Recall; PASi: Paired Associate Learning Immediate Recall; PASi_h: Hard Score of Paired Associate Learning Immediate Recall; SIM: similarities test; VRd: Visual Reproductions Delayed Recall; VRi: Visual Reproductions Immediate Recall; VRr: Visual Reproductions Recognition. Nodes with the same color represent NP tests measuring the same cognitive function.

Figure 4 shows the Bayesian network of hierarchical influence between NP tests and cognitive status. The network consists of nodes and edges, which represent the conditional dependence between NP tests and cognitive status. The parent node has an influence on the predictability of child nodes [49]. The first precursor of dementia is SIM, following the sequence of SIM–>BNT30–>VRi–>VRd, and eventually leading to dementia. On the other hand, the cognitive status could also influence visual memory indirectly via logical memory. Figure 5 shows Bayesian networks in subpopulations stratified by sex, ApoE, and education. For males, LMd (eg, verbal memory) directly influences the cognitive status, which then influences other NP tests. In other words, changes in verbal memory function are a precursor of dementia, which is consistent with the focus on memory as the key cognitive symptom of dementia [50]. The cognitive functions of visuospatial processing, visual memory, language, and verbal reasoning could also influence cognitive status. For females, the first precursor of dementia is SIM, following the sequence of SIM–>BNT30–>VRi–>VRd until the influencing cognitive status. Cognitive status also influences verbal memory and visual memory. Cognitive status influenced PASi indirectly via LMi. For participants carrying ApoE4 alleles, VRd influences cognitive status, followed by other NP tests. For participants without ApoE4 alleles, the relationship of NP tests and cognitive status is similar to the relationship among female only participants. For participants with low degrees of education, LMd influences cognitive status, which would then influence other NP tests. For participants with advanced degrees of education, VRd influences cognitive status and then LMi indirectly via PASi. VRr influences BNT30, which then influences SIM. It is worth noting that similar precursors of dementia were observed in females and participants without ApoE4 alleles. Gender-stratified ApoE4+ models can be found in Multimedia Appendix 1.

Taking the Bayesian network of females as an example (Figure 5), information is transmitted in a sequence of SIM–>BNT30–>VRi–>VRd–>Cognitive Status. If we have the VRd score, cognitive status becomes independent of SIM, BNT30, and VRi. Similarly, the block also exists in a diverging connection (LMi<–Cognitive Status–>PASi). Two child nodes, LMi and PASi, are related to each other by the Cognitive Status. However, if the participant is diagnosed with AD, LMi and PASi become conditionally independent. In other words, the decline of verbal memory and visual memory function does not influence each other. In the correlation network, all NP tests are related to cognitive status, but the hierarchical influence among NP tests and cognitive function cannot be distinguished. Some NP tests’ predictability of cognitive status is influenced by other NP tests.

The Markov blanket of cognitive status is the parent and child of the cognitive status node in Figure 4. Seven NP tests were included into Markov blankets of cognitive status for all Bayesian networks (Figure 5). The most frequent ones were LMi and VRd, which were parents of the cognitive status node in 4 and 5 Bayesian networks, respectively. The NP tests in Markov blankets have a direct first-level influence on cognitive status. As shown in Table 2, the degree of association between NP tests and cognitive status is declining when conditioned on the subset of Markov blanket. This reveals that the relationship between specific NP test and cognitive status is influenced by other tests. It provides a global and hierarchical view to understanding the relationship between NP tests and cognitive status. Future functional analyses can determine the specific role of these cognitive functions in AD pathogenesis.

The scores of NP tests were parameterized using probability tables as shown in Multimedia Appendix 1. The marginal probability and the probability dependency between NP tests were determined using the maximum likelihood estimation. Once the network was constructed and the probability was specified, Bayes theorem was used to propagate probability through the network to infer cognitive status. The performance of the model was evaluated using 10-fold cross validation. As shown in Multimedia Appendix 1, the overall accuracy was 82.2%, with sensitivity of 64.0% and specificity of 92.7%. The models stratified by sex, ApoE status, and education have similar performances.

**Table 2:** The *P* values of association between neuropsychological (NP) tests and cognitive status when conditioning on NP tests in Markov blanket. The significant degree was computed using Pearson chi-square test with adjusted degrees of freedom.

Neuropsychological tests	φ^a^, *P* value	VRd^b^, *P* value	LMi^c^, *P* value	VRd and LMi, *P* value
Logical Memory Delayed Recall	<.001	<.001	<.001	>.99
Logical Memory Recognition	<.001	<.001	.62	>.99
Visual Reproductions Immediate Recall	<.001	.18	<.001	>.99
Visual Reproductions Recognition	<.001	<.001	<.001	>.99
Boston Naming Test 30 item Even Version	<.001	<.001	<.001	>.99
Similarities test	<.001	<.001	<.001	>.99
Paired Associate Learning Immediate Recall	<.001	<.001	<.001	>.99
Hard Score of Paired Associate Learning Immediate Recall	<.001	<.001	<.001	>.99
Hard score of Paired Associate Learning Delayed Recall	<.001	<.001	<.001	>.99

^a^φ represents empty condition set.

^b^VRd: Visual Reproductions Delayed Recall.

^c^LMi: Logical Memory Immediate Recall.

### Alzheimer Disease Probabilistic Inference

We used Bayesian networks of total population to illustrate AD inferences, in which test scores were modeled as discrete intervals via clustering. Cognitive status was modeled by HC, AD, and NAD. SIM had no parent nodes, and their probabilities did not depend on other tests. Therefore, the probability for SIM was characterized by its marginal probabilities. For the tests influenced by others, conditional probabilities were used to reflect that relationship. The probabilities of a test can only be expressed by its immediate parent node. Although SIM influences cognitive status, only VRd was used in cognitive status’s conditional probability table (CPT). On the basis of the CPT, the prior probability of each NP test can be calculated. Influence between tests can also back-propagate along an edge. Using the Bayes theorem, we can compute the posteriors to infer cognitive status when NP tests are only partially available. Missing data from NP tests occur for various reasons, some of which are independent of cognitive status. This approach allows us to use the available information to make the best probabilistic inference, which does not depend on the same review sequence of NP tests. To reduce sampling effects, the inference process was repeated 100 times, and the mean of inference probability was used as the final clinical decision (Multimedia Appendix 1). To further validate whether the network structure can reflect the true relationship between NP tests and AD diagnosis, we simulated 3000 participants with 11 NP tests and correlated them with the cognitive status. As shown in Multimedia Appendix 1, all the relationships of simulated NP tests data were highly consistent with those derived from the original data. The discretized intervals derived from different populations are shown in Table 3 and Multimedia Appendix 1.

**Table 3:** Discretization of neuropsychological test scores.

Neuropsychological test	Score interval^a^
Logical Memory Immediate Recall	(0,3), (4,6), (7,8), (9,9), (10,10), (11,11), (12,12), (13,13), (14,16), (17,23)
Logical Memory Delayed Recall	(0,0), (1,3), (4,6), (7,8), (9,9), (10,10), (11,11), (12,12), (13,15), (16,24)
Logical Memory Recognition	(0,1), (2,3), (4,4), (5,5), (6,6), (7,7), (8,8), (9,9), (10,10), (11,11)
Visual Reproductions Immediate Recall	(0,2), (3,3), (4,4), (5,5), (6,6), (7,7), (8,8), (9,9), (10,10), (11,14)
Visual Reproductions Delayed Recall	(0,0), (1,1), (2,2), (3,3), (4,4), (5,5), (6,6), (7,7), (8,9), (10,14)
Visual Reproductions Recognition	(0,0), (1,1), (2,2), (3,3), (4,4)
Paired Associate Learning Immediate Recall	(0,7), (7.5,8.5), (9,9.5), (10,11), (11.5,12.5), (13,13.5), (14,14), (14.5,15.5), (16,17.5), (18,21)
Hard Score of Paired Associate Learning Immediate Recall	(0,0), (1,1), (2,2), (3,3), (4,4), (5,5), (6,6), (7,7), (8,8), (9,12)
Hard score of Paired Associate Learning Delayed Recall	(0,0), (1,1), (2,2), (3,3), (4,4)
Similarities Test	(0,6), (7,10), (11,12), (13,13), (14,14), (15,15), (16,16), (17,17), (18,19), (20,26)
Boston Naming Test 30 Item Even Version	(0,12), (13,17), (18,20), (21,23), (24,25), (26,26), (27,27), (28,28), (29,29), (30,30)

^a^Parentheses are used to indicate closed intervals, in case the square brackets are mistaken for references.

## Discussion

### Principal Findings

Various analytic models have been used to identify informative NP tests for AD prediction [4]. However, it is challenging to understand the hierarchical influence between NP tests. Our study explores the hierarchical probabilistic dependency of 11 NP tests and adjudicated cognitive outcomes—HC, AD, and NAD—based on the participants’ cognition at the time of their NP tests. These tests were incorporated into our Bayesian network to establish a probabilistic-based framework for cognitive outcomes. Within this theoretical hierarchical influence structure, upstream NP tests affect downstream NP tests, allowing us to probabilistically inference downstream NP tests. We observed interdependence between NP tests and overall cognition of an individual, where higher-level cognitive functions—represented by individual NP tests that preceded cognitive outcome in the hierarchy—are considered as precursors of dementia, whereas the lower-level cognitive functions are impacted as a result of dementia.

Machine learning methods have been used to predict cognitive decline [51,52]. The identification of preclinical patterns of cognitive decline is essential for the prevention and early treatment of AD [3,53,54]. NP tests have been used for AD diagnosis for a long time. Not surprisingly, all NP tests were found to be related to AD. However, the hierarchical relationship between NP tests was not previously fully investigated. Our data-driven findings depict the interplay of cognitive functions and further identify tests that influence, or are influenced by, cognitive status. Current use of cognitive tests largely focuses on declines that are in later disease states and thus cannot be used to detect preclinical disease states. We advocate that the decline of some cognitive functions is only the later manifestations of AD, not the precursor (ie, pre-AD performance metric). From a preventive medicine perspective, tests that are identified as precursors can be taken as reference for physicians to be considered as potential targets for AD intervention and perhaps prevention. The value of using patterns of NP tests is that they can be used as early screening tools to identify at-risk patients and provide interventions, including nonpharmacological therapies, which may delay or perhaps stop disease progression altogether.

Our study represents a significant step forward in how to better characterize preclinical AD heterogeneity. As cognitive functions tend to interrelate in complex ways, we explored conditional probability dependencies to present a main relationship between cognitive functions. For males, decline of memory function influences cognitive status, which also influences the functions of language, verbal reasoning, and visuospatial simultaneously. In comparison, for females, the relationships are more complex; verbal reasoning function influences language function, which influences visuospatial function and, subsequently, cognitive status. Cognitive status in females influences the function of logical and visual memory. It is noteworthy that although the interplay of cognitive functions has a different sequence for males vs females, logical memory and visual reproduction functions are typically the first precursors in AD. Our results suggest that gender-specific evaluations need to be considered by clinicians in AD clinics similar to other diseases such as heart attacks [55].

We also quantified functional connectivity through statistical correlations and coherence [56]. Functional connectivity is defined as a function of probability distributions over observed multivariate responses. The hierarchical influence among cognitive functions under various AD risk factors was assessed to understand psychological functional segregation for heterogeneous AD beyond just those associated with sex differences.

Our study has several advantages. In terms of method, the proposed structure of Bayesian network combined with inference offers several advantages in the inference of disease status. First, it provides a likelihood of the diagnosis, which is more intuitive and meaningful in a clinical setting [57,58]. In addition, we were able to impute the missing data by the probabilistic inference strategy, which was based on the network structure and training samples to capture the global assessment between NP tests. The long follow-up period, beginning in 1976, and the minimal loss to follow-up at FHS make it an ideal study population for AD research [59]. The AD diagnosis was adjudicated and verified by a panel consisting of at least one neurologist and one neuropsychologist; hence, outcome bias was minimized [3]. The NP tests within the FHS NP battery are well known and widely administered by many clinicians and researchers. Given these strengths, the results of this study can be readily translated into real-world application.

We also acknowledge several limitations of our study. The study participants are primarily of European ancestry, they have higher levels of education than the general population in the United States, and the majority of NP tests were carried out in English. In addition, the average age of dementia onset among FHS participants is around 85 years, which is higher than the expected average. Therefore, findings of our study might not be generalizable to other populations, such as these with lower educational attainment, other ancestries, or non-English–speaking groups. Moreover, we did not further distinguish participants with mild cognitive impairment from HCs. The models presented in this study were solely based on 11 NP tests from five categories of cognitive function. Introduction of other NP tests might reveal additional interactions between cognitive functions and help to strengthen the overall model.

### Conclusions

We developed a probabilistic dependency network for AD diagnosis from 11 NP tests. Our study revealed important psychological functional segregations and precursor evidence of AD development. Future validations with additional samples and NP tests would provide a more comprehensive picture of cognitive function and identify potential NP biomarkers for AD surveillance.

## Appendix

Multimedia Appendix 1 Details of methods and supplement results.

## Abbreviations

AD: Alzheimer disease
ApoE4: apolipoprotein E4
BNT30: Boston Naming Test 30 item Even Version
CPT: conditional probability table
FHS: Framingham Heart Study
HC: healthy control
LMi: Logical Memory Immediate Recall
LMd: Logical Memory Delayed Recall
NAD: non-Alzheimer dementia
NP: neuropsychological
PASi: Paired Associate Learning Immediate Recall
SIM: similarities test
VRi: Visual Reproductions Immediate Recall
VRd: Visual Reproductions Delayed Recall
VRr: Visual Reproductions Recognition
