# Supplementary material for: Exploring the Hierarchical Influence of Cognitive Functions for Alzheimer Disease: The Framingham Heart Study
Source: J Med Internet Res. 2020 Apr 23;22(4):e15376. doi: 10.2196/15376 (PMC7206516; doi:10.2196/15376)

Supplemental Materials

Supplemental Methods

Partitioning Around Medoids for Discretization

The partitioning around medoids (PAM) [1] is based on the search for k (from 1 to 10) representative medoids of NP test scores. By assigning each participant to the nearest medoid, k clusters are constructed after finding a group of medoids. The goal is to select k representative cutoff scores which minimize the summation of the dissimilarities of the participants to their closest representative medoids which is calculated by Euclidean distance. We determined the number of homogeneous clusters based on optimum average silhouette width which ranging from 0 to 1 [2]. The greater the silhouette width is, the better the clustering performance obtained. A silhouette width greater than 0.7 indicates a very strong structure in the classification. The silhouette width ranging between 0.5 and 0.7 indicates a reasonable structure [3].

Structure Learning

The heuristic hill-climbing greedy search for the best candidate network structure starts from an empty structure which only had 12 nodes (corresponding to 11 NP tests and cognitive status) with no arc. The initial BIC score was -107289.3. Then the algorithm attempted to add one arc between 12 nodes, which in total consisted of $A_{12}^{2}=132$ arcs. We also tried to remove and reverse one of the arcs whose number was 0 at each current step. At the same time, the algorithm computed the BIC scores of the candidate Bayesian networks (BNs) generated by the above operations. The best operation added PASi→PASi_h with the biggest BIC score equal to -101087.7. Then a new BN structure was obtained with 12 nodes and 1 arc, which was PASi→PASi_h. We tried adding one of 130 (A_{12}^{2}-2) arcs, removing one of 1 arcs and reversing one of 1 arcs. The best operation added LMi→LMd with the biggest BIC score - 97941.68. The algorithm attempted every possible operation in an iterating loop, making the network with the highest BIC score as candidate structure of BN. The operations included single-edge addition, removal, or reversal. The algorithm stopped when changing one edge did not increase the BIC score of network structure. The optimal BN structure obtained had a BIC score of -87952.39.

To minimize the uncertainty of the model, bootstrap was adapted to resample the data 200 times. Then algorithm was run on these datasets to constructed 200 network structures [4]. The frequency of each arc that appears in those 200 networks was computed. Consensus network was constructed by picking up arcs whose frequencies are above a threshold. The value 0.7 was selected because it will produce a sparse network that is easier to interpret. Besides the better prediction performance, the averaged Bayesian network model is also less affected by noisy data.

Parameters Learning

The maximum likelihood estimation tries to learn parameters to maximize the likelihood function. The maximum likelihood principle is to choose the unknown parameters which could ensure that the overall probability of the training data is maximized.

Supplemental Materials

The probability of the training data which has T participants and 11 NP tests is shown in **Equation 1**. To make parameters learning feasible, we made assumption that each participant is independent and identically distributed (iid) from the joint distribution defined by the BNs. With this assumption, the probability of participant t is shown in **Equation 2**. $Pa(X_i)$ represents the parent of node X_i in the BN. Then we let w represent these parameters. Based on [5], the principle says that we should choose $w^* = \arg\max_w P$, because the logarithm function is strictly increasing monotonically, which is equivalent to choosing $w^* = \arg\max_w \log P$. So the goal is to maximize **Equation 3**.

$$P=\prod_{t=1}^{T} p(X_{LMd} = x_{t1},...,X_{BNT30} = x_{t11}) \quad (1)$$

$$p(X_{LMd} = x_{t1},...,X_{BNT30} = x_{t11}) = \prod_{i=1}^{11} p(X_i = x_{ti} \mid pa(X_i) = pa_{ti}) \quad (2)$$

$$Loss = \log \prod_{t=1}^{T}\prod_{i=1}^{11} p(X_i = x_{ti} \mid pa(X_i) = pa_{ti}) = \sum_{t=1}^{T}\sum_{i=1}^{11} \log p(X_i = x_{ti} \mid pa(X_i) = pa_{ti}) \quad (3)$$

Equation 4 is obtained after swapping the order of the summations**.**

$$Loss = \sum_{i=1}^{11}\sum_{t=1}^{T} \log p(X_i = x_{ti} \mid pa(X_i) = pa_{ti}) \quad (4)$$

In other words, assuming that the parent of X_i is π, the maximum likelihood estimate of the probability of X_i = x is shown in **Equation 5**, where the counts are relative to the training data.

$$p(X_i = x \mid pa(X_i) = \pi) = \frac{count(X_i = x, pa(X_i) = \pi)}{\sum_{x'} count(X_i = x', pa(X_i) = \pi)} = \frac{count(X_i = x, pa(X_i) = \pi)}{count(pa(X_i) = \pi)} = \frac{\sum_t I(x = x_{ti}, \pi = pa_{ti})}{\sum_t I(\pi = pa_{ti})} \quad (5)$$

Each estimated probability is proportional to the corresponding frequency in the training data. If the value x is never observed for some combination π, then its conditional probability is estimated to be 0. Below is an example. In the BN of total population, SIM has no parents, so here is the probability of SIM score equals to1

$$p(SIM = 1) = \frac{count(SIM = 1)}{\sum_{i=1}^{10} count(SIM = i)} = \frac{310}{4512} \approx 0.0687 \quad (6)$$

BNT30 has parent SIM. So when the SIM score equals to 1, 0.1742 is the probability of BNT30 score equals to 1.

$$p(BNT30 = 1 \mid SIM = 1) = \frac{count(BNT30 = 1,\ SIM = 1)}{\sum_{i=1}^{10} count(BNT30 = i, SIM = 1)} = \frac{count(BNT30 = 1, SIM = 1)}{count(SIM = 1)} = \frac{54}{310} \approx 0.1742 \quad (7)$$

All parameters in conditional probability tables are calculated in this manner.

AD Inference

The objective of averaging likelihood weighting simulation is to calculate the posterior probabilities of cognitive status, given some observed scores from NP tests. It generates a set of randomly selected participants (n = 1000) based on the structure and parameter of BN, and then approximate probabilities of cognitive status by the frequencies of appearances in the simulated participants. When sampling the simulated participants, the scores of some NP tests will be known while others not due to missing

data or time limit of assessment that didn't allow administration of some tests. The NP tests with scores are referred to as evidence. Given the evidence, we need to query the remaining nodes in BN to determine the cognitive status, the sampling process is as follows.

We set a temporary variable w = 1 which holds the calculated weight of the simulated participant. A temporary variable, x is set to empty. If the pending participant has 3 NP tests (LMd = 2, VRd = 3, PASi = 3) and we want to infer whether this person has AD, this available information could be represented as x = {LMd = 2, VRd = 3, PASi = 3, Cognitive Status=AD}. Each NP test node in the BN is examined. If the score from a NP test is available, then we make the following calculation: $w = w \times$ P(current NP test | parents of current NP test). If the current NP test score is unknown, then it is sampled to determine its value. It does not contribute to the weight calculation. Whether the available NP test is considered as evidence, or whether the missing NP test is discovered through sampling, its state is added to x. After the entire network is examined for this participant, we will be left with x and w, representing the status of the participant and the likelihood weight, respectively. This is added to W using x as the simulated participant, w as the corresponding weight. If x already exists in W, then w is added to weight associated to x in W.

Take the part of BN as an example. For this pending participant with known information SIM = 2, VRi = 2, we will generate 10000 simulated participants to determine his cognitive status. We now set the weight w = 1.0 and $x = \{\varnothing\}$. SIM is evidence with a score of 2. Therefore, we set $w = w \times$ P(SIM = 2) = (1.0) × (0. 114444) = 0. 114444, x = {SIM = 2}. BNT30 is not available. So we sample from P(BNT30 | SIM = 2) = <0.04277, 0.175153, 0.173116, 0.197556, 0.193483, 0.08554, 0.046843, 0.05499, 0.01833, 0.01222>. Suppose this returns BNT30 = 3. x = {SIM = 2, BNT30 = 3}. VRi is evidence with a score of 2. We set $w = w \times$ P(VRi = 2 | BNT30 = 3) = (0. 114444) × (0.171779) = 0.01965. x = {SIM = 2, BNT30 = 3, VRi = 2}. VRd is not available. So we sample from P(VRd | VRi = 2) = <0.128378, 0.186937, 0.27027, 0.277027, 0.094595, 0.027027, 0.013514, 0.002252, 0, 0> Suppose this returns VRd = 3. x = {SIM = 2, BNT30 = 3, VRi = 2, VRd = 3}. Then cognitive status is not available. So we sample from P (Cognitive Status | VRd = 3) = < 0.633411, 0.185615, 0.180974>. Suppose this returns Cognitive Status = AD. x = {SIM = 2, BNT30 = 3, VRi = 2, VRd = 3, Cognitive Status = AD}. The belief updating follows the path of BN. Finally, based on the above we now add {SIM = 2, BNT30 = 3, VRi = 2, VRd = 3, Cognitive Status = AD} to W with a weight of 0.01965. Then the sampling process is repeated until there are 10000 simulated participants.

Supplemental Materials

Reference

1 Reynolds A P, Richards G, de la Iglesia B, et al. Clustering rules: a comparison of partitioning and hierarchical clustering algorithms. Journal of Mathematical Modelling and Algorithms 2006 Mar, 5(4): 475-504. [doi:10.1007/s10852-005-9022-1]

2 Kaufman L, Rousseeuw PJ. Finding groups in data: an introduction to cluster analysis: John Wiley & Sons; 2005. ISBN: 0471735787

3 Arunajadai SG. RNA Unwinding by NS3 Helicase: A Statistical Approach. Plos One 2009 Sep 22;4(9). [doi: 10.1371/journal.pone.0006937]

4 Friedman N, Linial M, Nachman I, Pe'er D. Using Bayesian networks to analyze expression data. J Comput Biol 2000;7(3-4):601-20. [doi: 10.1089/106652700750050961]

5 Silvey SD. Statistical inference. eBook. Routledge; 2017. ISBN: 0412138204

Supplemental Materials

Supplemental Results

eTable 1. Summary statistics of all available 32 NP tests in FHS.

NP Tests[a]	Acronyms	Range	Mean (SD)	Missing (%)
Logical Memory – IR	LMi	0–23	10.55 (4.53)	244 (2%)
Logical Memory – DR	LMd	0–23	9.52 (4.74)	335 (2%)
Logical Memory – Recognition	LMr	0–11	9.29 (1.59)	2241 (15%)
Visual Reproductions – IR	VRi	0–14	7.57 (3.70)	586 (4%)
Visual Reproductions – DR	VRd	0–14	6.74 (3.88)	685 (4%)
Visual Reproductions – Recognition	VRr	0–4	2.72 (1.23)	752 (5%)
Paired Associate Learning – IR	PASi	0–21	13.15 (4.06)	743 (5%)
Paired Associate Learning – IR (Ease Score)	PASi_e	0–18	16.29 (2.43)	741 (5%)
Paired Associate Learning – IR (Hard Score)	PASi_h	0–12	4.91 (3.37)	743 (5%)
Paired Associate Learning – DR	PASd	0–10	8.14 (1.76)	2452 (16%)
Paired Associate Learning – DR (Ease Score)	PASd_e	0–6	5.80 (0.65)	2452 (16%)
Paired Associate Learning – DR (Hard Score)	PASd_h	0–4	2.34 (1.40)	2451 (16%)
Paired Associate Learning - Recognition	PASr	0–10	9.65 (1.19)	7360 (48%)
Digits Forward Span	DSF	0–9	6.42 (1.39)	3314 (22%)
Digits Backward Span	DSB	0–8	4.58 (1.40)	3480 (23%)
Trail A[b]	trailsA	0–7	0.66 (0.64)	2642 (17%)
Trail B[b]	trailsB	0–10	1.98 (2.07)	2995 (19%)
Similarities Test	SIM	0–26	15.20 (5.14)	500 (3%)
Hooper Visual Organization Test	HVOT	0–30	24.18 (4.47)	2768 (18%)
Boston Naming test – 10 items	BNT10	0–10	9.25 (1.46)	661 (4%)
Boston Naming test – 10 items (semantic cue)	BNT10_semantic	0–4	0.08 (0.29)	2446 (16%)
Boston Naming test – 10 items (phonemic cue)	BNT10_phonemic	0–4	0.21 (0.50)	2446 (16%)
Boston Naming test – 30 items	BNT30	0–30	26.06 (4.80)	2447 (16%)
Boston Naming test – 30 items (semantic cue)	BNT30_semantic	0–6	0.35 (0.66)	2447 (16%)
Boston Naming test – 30 items (phonemic cue)	BNT30_phonemic	0–15	1.23 (1.43)	2447 (16%)
Finger Tapping – Right hand	FingTapR	0–77.6	45.63 (10.30)	5835 (38%)
Finger Tapping – Left hand	FingTapL	0–74	41.49 (9.12)	5835 (38%)
Wide Range Achievement Test –Reading	WRAT	15–57	48.80 (5.35)	3424 (22%)
Verbal Fluency Test	FAS	0–95	35.00 (14.44)	3667 (24%)
Verbal Fluency Test – Animal	FAS_animal	0–50	18.61 (6.05)	7415 (48%)
Block Design	BD	0–26	19.87 (6.34)	10716 (70%)
WAIS test	WAIS	0–29	17.89 (6.17)	10616 (69%)

[a] IR: Immediate Recall; DR: Delayed Recall.

[b] Measured in minutes.

Supplemental Materials

eTable 2. The conditional probability table of NP tests and cognitive status for the total population learned by the maximum likelihood estimation.

Node LMi

P(LMi |Cognitive Status)	Cognitive Status = AD	Cognitive Status = NAD	Cognitive Status = HC
1	0.02	0.43	0.13
2	0.08	0.26	0.25
3	0.12	0.15	0.18
4	0.09	0.04	0.10
5	0.10	0.03	0.08
6	0.11	0.02	0.06
7	0.11	0.03	0.07
8	0.10	0.01	0.06
9	0.20	0.02	0.06
10	0.07	0.01	0.01

Node LMd

P(LMd|LMi)	1	2	3	4	5	6	7	8	9	10
1	0.62	0.13	0.05	0.01	0.00	0.00	0.00	0.00	0.00	0.00
2	0.35	0.28	0.05	0.01	0.01	0.00	0.00	0.00	0.00	0.00
3	0.03	0.46	0.39	0.19	0.09	0.04	0.01	0.01	0.00	0.00
4	0.01	0.11	0.36	0.37	0.25	0.16	0.08	0.02	0.02	0.00
5	0.00	0.01	0.08	0.22	0.23	0.18	0.12	0.05	0.02	0.00
6	0.00	0.01	0.05	0.09	0.20	0.24	0.18	0.14	0.04	0.00
7	0.00	0.00	0.02	0.07	0.13	0.19	0.26	0.15	0.08	0.01
8	0.00	0.00	0.01	0.03	0.07	0.11	0.17	0.20	0.15	0.03
9	0.00	0.00	0.00	0.01	0.03	0.07	0.17	0.42	0.53	0.28
10	0.00	0.00	0.00	0.00	0.00	0.00	0.00	0.02	0.15	0.67

Node LMr

P(LMr|LMd)	1	2	3	4	5	6	7	8	9	10
1	0.03	0.00	0.00	0.00	0.00	0.00	0.00	0.00	0.00	0.00
2	0.08	0.02	0.00	0.00	0.00	0.00	0.00	0.00	0.00	0.00
3	0.13	0.04	0.00	0.00	0.00	0.00	0.00	0.00	0.00	0.00
4	0.15	0.09	0.02	0.00	0.01	0.00	0.00	0.00	0.00	0.00
5	0.21	0.18	0.05	0.02	0.01	0.00	0.00	0.00	0.00	0.00
6	0.14	0.23	0.18	0.10	0.07	0.03	0.02	0.02	0.01	0.00
7	0.15	0.22	0.27	0.16	0.15	0.11	0.11	0.07	0.03	0.01
8	0.07	0.14	0.25	0.34	0.28	0.28	0.27	0.20	0.18	0.08
9	0.04	0.07	0.18	0.28	0.33	0.39	0.39	0.39	0.43	0.34
10	0.00	0.01	0.04	0.09	0.16	0.18	0.21	0.32	0.35	0.57

Node VRi

P(VRi|BNT30)	1	2	3	4	5	6	7	8	9	10
1	0.71	0.53	0.32	0.17	0.11	0.08	0.04	0.03	0.03	0.01
2	0.11	0.20	0.17	0.17	0.13	0.10	0.05	0.05	0.03	0.02
3	0.03	0.11	0.15	0.18	0.12	0.11	0.09	0.09	0.03	0.02
4	0.03	0.09	0.14	0.13	0.15	0.09	0.12	0.10	0.08	0.05
5	0.06	0.03	0.08	0.12	0.12	0.15	0.10	0.13	0.10	0.09
6	0.01	0.02	0.05	0.07	0.11	0.12	0.12	0.12	0.12	0.11
7	0.01	0.00	0.05	0.06	0.07	0.13	0.11	0.13	0.14	0.15

8	0.01	0.00	0.02	0.05	0.07	0.08	0.12	0.12	0.13	0.11
9	0.00	0.01	0.01	0.02	0.05	0.07	0.09	0.09	0.13	0.14
10	0.02	0.00	0.01	0.02	0.06	0.08	0.14	0.12	0.22	0.29
Node VRd										
P(VRd|VRi)	1	2	3	4	5	6	7	8	9	10
1	0.43	0.13	0.05	0.04	0.01	0.02	0.00	0.00	0.00	0.00
2	0.27	0.19	0.12	0.04	0.03	0.02	0.01	0.01	0.00	0.00
3	0.20	0.27	0.20	0.10	0.06	0.02	0.02	0.01	0.00	0.00
4	0.07	0.28	0.21	0.16	0.10	0.06	0.03	0.02	0.02	0.00
5	0.02	0.09	0.27	0.21	0.14	0.11	0.05	0.03	0.02	0.01
6	0.00	0.03	0.10	0.30	0.21	0.15	0.11	0.08	0.05	0.01
7	0.00	0.01	0.02	0.10	0.30	0.17	0.16	0.10	0.04	0.03
8	0.00	0.00	0.01	0.04	0.10	0.28	0.22	0.18	0.10	0.05
9	0.00	0.00	0.00	0.01	0.04	0.16	0.37	0.42	0.32	0.16
10	0.00	0.00	0.00	0.00	0.00	0.01	0.04	0.15	0.46	0.73
Node VRr										
P(VRr|VRd)	1	2	3	4	5	6	7	8	9	10
1	0.34	0.17	0.16	0.07	0.08	0.04	0.01	0.01	0.01	0.00
2	0.36	0.37	0.35	0.31	0.21	0.18	0.14	0.08	0.05	0.02
3	0.23	0.31	0.31	0.33	0.34	0.31	0.29	0.29	0.18	0.09
4	0.05	0.14	0.14	0.22	0.29	0.34	0.39	0.41	0.40	0.27
5	0.02	0.00	0.04	0.07	0.08	0.13	0.17	0.21	0.36	0.62
Node PASi										
P(PASi|LMd)	1	2	3	4	5	6	7	8	9	10
1	0.50	0.24	0.08	0.05	0.05	0.01	0.02	0.01	0.01	0.00
2	0.25	0.22	0.15	0.08	0.09	0.06	0.08	0.06	0.04	0.01
3	0.10	0.13	0.16	0.13	0.12	0.08	0.08	0.08	0.05	0.02
4	0.09	0.23	0.25	0.24	0.21	0.20	0.17	0.15	0.14	0.04
5	0.03	0.09	0.15	0.15	0.16	0.13	0.13	0.14	0.10	0.12
6	0.01	0.03	0.07	0.11	0.11	0.15	0.12	0.10	0.12	0.09
7	0.01	0.03	0.05	0.07	0.07	0.10	0.07	0.07	0.06	0.08
8	0.00	0.02	0.05	0.08	0.07	0.10	0.10	0.13	0.11	0.12
9	0.01	0.01	0.04	0.07	0.06	0.11	0.14	0.15	0.19	0.23
10	0.00	0.00	0.01	0.02	0.06	0.05	0.09	0.11	0.16	0.29
Node PASi_h										
P(PASi_h|PASi)	1	2	3	4	5	6	7	8	9	10
1	0.90	0.67	0.29	0.00	0.00	0.00	0.00	0.00	0.00	0.00
2	0.09	0.28	0.45	0.15	0.00	0.00	0.00	0.00	0.00	0.00
3	0.01	0.04	0.23	0.53	0.00	0.00	0.00	0.00	0.00	0.00
4	0.00	0.01	0.04	0.29	0.54	0.00	0.00	0.00	0.00	0.00
5	0.00	0.00	0.00	0.04	0.40	0.50	0.00	0.00	0.00	0.00
6	0.00	0.00	0.00	0.00	0.05	0.45	0.73	0.00	0.00	0.00
7	0.00	0.00	0.00	0.00	0.00	0.04	0.25	0.68	0.00	0.00
8	0.00	0.00	0.00	0.00	0.00	0.00	0.01	0.30	0.39	0.00
9	0.00	0.00	0.00	0.00	0.00	0.00	0.00	0.03	0.51	0.00
10	0.00	0.00	0.00	0.00	0.00	0.00	0.00	0.00	0.11	1.00
Node PASd_h										

Supplemental Materials

P(PASd_h|PASi_h)	1	2	3	4	5	6	7	8	9	10
1	0.84	0.49	0.23	0.11	0.07	0.03	0.01	0.00	0.00	0.00
2	0.15	0.40	0.49	0.37	0.27	0.19	0.09	0.04	0.00	0.01
3	0.01	0.10	0.23	0.39	0.41	0.42	0.31	0.24	0.18	0.06
4	0.00	0.01	0.05	0.12	0.23	0.27	0.41	0.42	0.37	0.19
5	0.00	0.00	0.01	0.01	0.03	0.08	0.18	0.29	0.45	0.75
Node BNT30										
P(BNT30|SIM)	1	2	3	4	5	6	7	8	9	10
1	0.17	0.04	0.01	0.00	0.01	0.00	0.00	0.00	0.00	0.00
2	0.26	0.18	0.07	0.05	0.02	0.01	0.02	0.01	0.00	0.00
3	0.20	0.17	0.12	0.10	0.07	0.04	0.03	0.03	0.02	0.00
4	0.19	0.20	0.22	0.25	0.15	0.15	0.10	0.08	0.06	0.02
5	0.09	0.19	0.22	0.21	0.27	0.23	0.19	0.17	0.12	0.07
6	0.04	0.09	0.11	0.12	0.13	0.13	0.12	0.09	0.10	0.05
7	0.03	0.05	0.09	0.10	0.14	0.15	0.14	0.15	0.16	0.12
8	0.01	0.05	0.07	0.09	0.10	0.12	0.17	0.20	0.15	0.15
9	0.01	0.02	0.05	0.06	0.08	0.12	0.13	0.17	0.21	0.27
10	0.00	0.01	0.04	0.02	0.05	0.05	0.10	0.10	0.18	0.31
Node Cognitive Status										
P(Cognitive Status|VRd)	1	2	3	4	5	6	7	8	9	10
1	0.20	0.41	0.63	0.68	0.84	0.88	0.93	0.96	0.96	0.98
2	0.60	0.34	0.19	0.14	0.04	0.06	0.02	0.01	0.02	0.00
3	0.20	0.24	0.18	0.17	0.11	0.06	0.04	0.03	0.03	0.01
Node SIM										
P(SIM)	1	2	3	4	5	6	7	8	9	10
[a]Freq	0.07	0.11	0.10	0.09	0.08	0.09	0.09	0.10	0.16	0.11

[a]Freq represents the frequency of corresponding values in the training data.

Supplemental Materials

eTable 3. The performance of BNs derived from different subpopulations.

		Maximum Likelihood Estimation			Bayesian Parameter Estimation		
		Accuracy	Sensitivity	Specificity	Accuracy	Sensitivity	Specificity
Total		82.2%	64.0%	92.7%	82.1%	62.9%	93.0%
Sex	Male	80.4%	64.9%	89.6%	80.3%	64.4%	89.6%
	Female	82.1%	63.0%	92.5%	82.0%	63.3%	92.3%
ApoE4 allele[a]	ApoE4 -	83.9%	60.9%	94.1%	83.8%	60.7%	94.1%
	ApoE4 +	76.2%	72.1%	89.1%	76.0%	70.6%	88.8%
Education	High school and below	78.6%	72.3%	89.7%	78.2%	71.8%	89.1%
	Beyond high school	84.5%	47.1%	95.1%	84.0%	45.7%	94.9%

[a]ApoE4 -: ApoE genotype 2/2, 2/3 or 3/3; ApoE4 +: ApoE genotype 3/4 or 4/4.

Participants who did not consent to genetic analyses, had an ApoE2/4 genotype or with no ApoE e4 information were excluded.

Supplemental Materials

eTable 4. Discretization of NP test scores in males.

NP test	Score interval
LMi	[0,3],[4,6],[7,8],[9,9],[10,10],[11,11],[12,12],[13,13],[14,15],[16,23]
LMd	[0,1],[2,5],[6,7],[8,8],[9,9],[10,10],[11,11],[12,12],[13,14],[15,24]
LMr	[0,0],[2,3],[4,4],[5,5],[6,6],[7,7],[8,8],[9,9],[10,10],[11,11]
VRi	[0,2],[3,3],[4,4],[5,5],[6,6],[7,7],[8,8],[9,9],[10,11],[12,14]
VRd	[0,1],[2,2],[3,3],[4,4],[5,5],[6,6],[7,7],[8,8],[9,10],[11,14]
VRr	[0,0],[1,1],[2,2],[3,3],[4,4]
PASi	[0,7],[7.5,8.5],[9,9],[9.5,10.5],[11,11.5],[12,13],[13.5,14],[14.5,15],[15.5,17],[17.5,21]
PASi_h	[0,0],[1,1],[2,2],[3,3],[4,4],[5,5],[6,6],[7,7],[8,8],[9,12]
PASd_h	[0,0],[1,1],[2,2],[3,3],[4,4]
SIM	[0,6],[7,11],[12,13],[14,14],[15,15],[16,16],[17,17],[18,18],[19,19],[20,26]
BNT30	[0,14],[15,19],[20,22],[23,24],[25,25],[26,26],[27,27],[28,28],[29,29],[30,30]

Supplemental Materials

eTable 5. Discretization of NP test scores in females.

NP test	Score interval
LMi	[0,1],[2,4],[5,7],[8,9],[10,10],[11,11],[12,12],[13,13],[14,16],[17,23]
LMd	[0,1],[2,4],[5,7],[8,9],[10,10],[11,11],[12,12],[13,13],[14,15],[16,24]
LMr	[0,1],[2,3],[4,4],[5,5],[6,6],[7,7],[8,8],[9,9],[10,10],[11,11]
VRi	[0,1],[2,2],[3,3],[4,4],[5,5],[6,6],[7,7],[8,8],[9,10],[11,14]
VRd	[0,0],[1,1],[2,2],[3,3],[4,4],[5,5],[6,6],[7,7],[8,9],[10,14]
VRr	[0,0],[1,1],[2,2],[3,3],[4,4]
PASi	[0,7.5],[8,9],[9.5,10],[10.5,11.5],[12,12.5],[13,13],[13.5,14.5],[15,16],[16.5,17.5],[18,21]
PASi_h	[0,0],[1,1],[2,2],[3,3],[4,4],[5,5],[6,6],[7,7],[8,8],[9,12]
PASd_h	[0,0],[1,1],[2,2],[3,3],[4,4]
SIM	[0,5],[6,9],[10,11],[12,13],[14,14],[15,15],[16,16],[17,17],[18,19],[20,26]
BNT30	[0,6],[7,15],[16,19],[20,22],[23,24],[25,25],[26,26],[27,27],[28,28],[29,30]

Supplemental Materials

eTable 6. Discretization of NP test scores in participants without ApoE4 alleles.

NP test	Score interval
LMi	[0,4],[5,7],[8,9],[10,10],[11,11],[12,12],[13,13],[14,14],[15,16],[17,23]
LMd	[0,1],[2,4],[5,7],[8,8],[9,9],[10,10],[11,11],[12,13],[14,15],[16,24]
LMr	[0,1],[2,3],[4,4],[5,5],[6,6],[7,7],[8,8],[9,9],[10,10],[11,11]
VRi	[0,2],[3,3],[4,4],[5,5],[6,6],[7,7],[8,8],[9,9],[10,11],[12,14]
VRd	[0,0],[1,1],[2,2],[3,3],[4,4],[5,5],[6,6],[7,7],[8,9],[10,14]
VRr	[0,0],[1,1],[2,2],[3,3],[4,4]
PASi	[0,7],[7.5,8],[8.5,9.5],[10,11],[11.5,12.5],[13,13.5],[14,14],[14.5,15.5],[16,17.5],[18,21]
PASi_h	[0,0],[1,1],[2,2],[3,3],[4,4],[5,5],[6,6],[7,7],[8,8],[9,12]
PASd_h	[0,0],[1,1],[2,2],[3,3],[4,4]
SIM	[0,7],[8,11],[12,13],[14,14],[15,15],[16,16],[17,17],[18,18],[19,20],[21,26]
BNT30	[0,16],[17,20],[21,22],[23,24],[25,25],[26,26],[27,27],[28,28],[29,29],[30,30]

Supplemental Materials

eTable 7. Discretization of NP test scores in participants with ApoE4 alleles.

NP test	Score interval
LMi	[0,2],[3,5],[6,7],[8,8],[9,9],[10,10],[11,11],[12,13],[14,16],[17,21]
LMd	[0,0],[1,3],[4,6],[7,8],[9,9],[10,10],[11,12],[13,13],[14,15],[16,20]
LMr	[0,0],[2,3],[4,4],[5,5],[6,6],[7,7],[8,8],[9,9],[10,10],[11,11]
VRi	[0,1],[2,2],[3,3],[4,4],[5,5],[6,6],[7,7],[8,8],[9,10],[11,14]
VRd	[0,0],[1,1],[2,2],[3,3],[4,4],[5,5],[6,6],[7,7],[8,9],[10,13]
VRr	[0,0],[1,1],[2,2],[3,3],[4,4]
PASi	[0,2],[3,6],[6.5,7],[7.5,8.5],[9,10],[10.5,11.5],[12,13],[13.5,14.5],[15,16],[16.5,21]
PASi_h	[0,0],[1,1],[2,2],[3,3],[4,4],[5,5],[6,6],[7,7],[8,8],[9,12]
PASd_h	[0,0],[1,1],[2,2],[3,3],[4,4]
SIM	[0,5],[6,9],[10,11],[12,13],[14,14],[15,15],[16,16],[17,17],[18,20],[21,25]
BNT30	[0,7],[9,18],[19,21],[22,23],[24,24],[25,25],[26,26],[27,27],[28,28],[29,30]

Supplemental Materials

eTable 8. Discretization of NP test scores in participants with high school and below degree.

NP test	Score interval
LMi	[0,2],[3,5],[6,7],[8,8],[9,9],[10,10],[11,11],[12,12],[13,14],[15,22]
LMd	[0,1],[2,4],[5,6],[7,7],[8,8],[9,9],[10,10],[11,11],[12,13],[14,23]
LMr	[0,2],[3,3],[4,4],[5,5],[6,6],[7,7],[8,8],[9,9],[10,10],[11,11]
VRi	[0,1],[2,2],[3,3],[4,4],[5,5],[6,6],[7,7],[8,8],[9,10],[11,14]
VRd	[0,0],[1,1] [2,2],[3,3],[4,4],[5,5],[6,6],[7,7],[8,9],[10,14]
VRr	[0,0],[1,1],[2,2],[3,3],[4,4]
PASi	[0,5.5],[6,7.5],[8,8.5],[9,10],[10.5,11.5],[12,12],[12.5,13.5],[14,14.5],[15,16.5],[17,21]
PASi_h	[0,0],[1,1],[2,2],[3,3],[4,4],[5,5],[6,6],[7,7],[8,8],[9,12]
PASd_h	[0,0],[1,1],[2,2],[3,3],[4,4]
SIM	[0,3],[4,6],[7,9],[10,11],[12,12],[13,13],[14,14],[15,15],[16,17],[18,23]
BNT30	[0,6],[7,15],[16,19],[20,22],[23,24],[25,25],[26,26],[27,27],[28,28],[29,30]

Supplemental Materials

eTable 9. Discretization of NP test scores in the participants with beyond high school degree.

NP test	Score interval
LMi	[0,4],[5,7],[8,9],[10,10],[11,11],[12,12],[13,13],[14,14],[15,16],[17,23]
LMd	[0,2],[3,6],[7,8],[9,9],[10,10],[11,11],[12,12],[13,13],[14,15],[16,21]
LMr	[0,0],[2,3],[4,4],[5,5],[6,6],[7,7],[8,8],[9,9],[10,10],[11,11]
VRi	[0,2],[3,3],[4,4],[5,5],[6,6],[7,7],[8,8],[9,9],[10,10],[11,14]
VRd	[0,1],[2,2],[3,3],[4,4],[5,5],[6,6],[7,7],[8,8],[9,10],[11,14]
VRr	[0,0],[1,1],[2,2],[3,3],[4,4]
PASi	[0,8],[8.5,9],[9.5,10.5],[11,11.5],[12,13],[13.5,14.5],[15,16],[16.5,17],[17.5,18],[18.5,21]
PASi_h	[0,0],[1,1],[2,2],[3,3],[4,4],[5,5],[6,6],[7,7],[8,8],[9,12]
PASd_h	[0,0],[1,1],[2,2],[3,3],[4,4]
SIM	[0,7],[8,11],[12,13],[14,14],[15,15],[16,16],[17,17],[18,18],[19,20],[21,26]
BNT30	[0,16],[17,20],[21,23],[24,24],[25,25],[26,26],[27,27],[28,28],[29,29],[30,30]

Supplemental Materials

eFigure 1. The visualization of Pearson's correlation coefficients between NP tests in subpopulations.

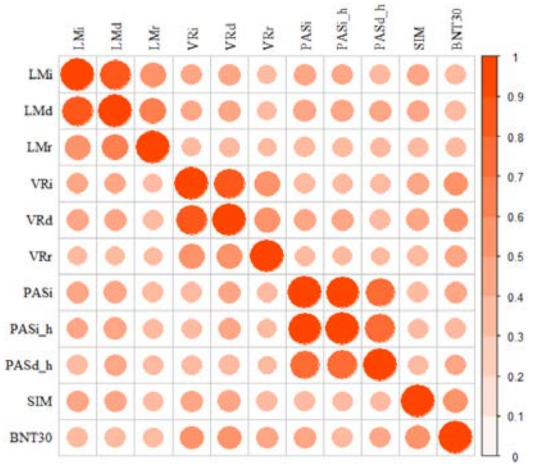

Male

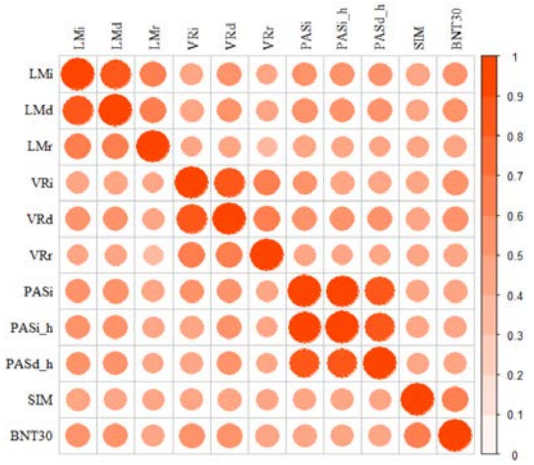

Female

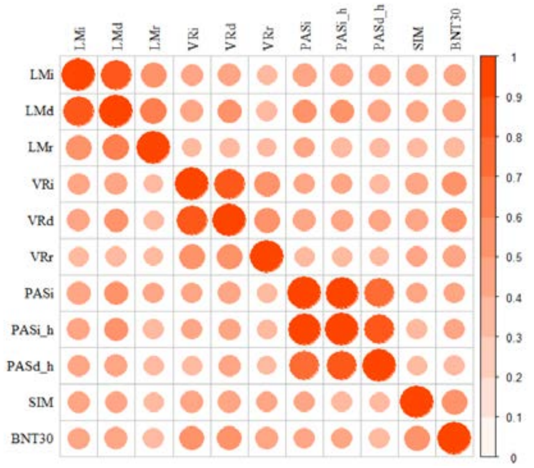

ApoE4 -

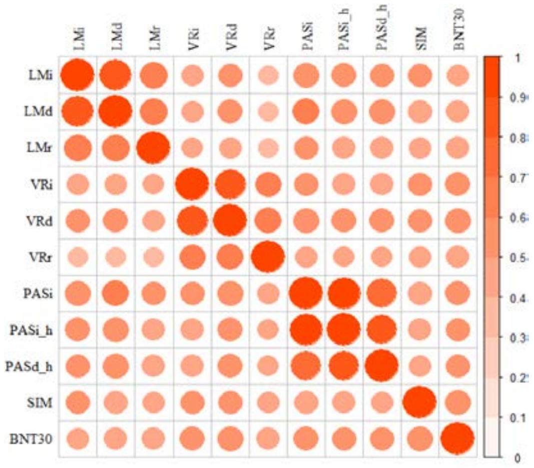

ApoE4 +

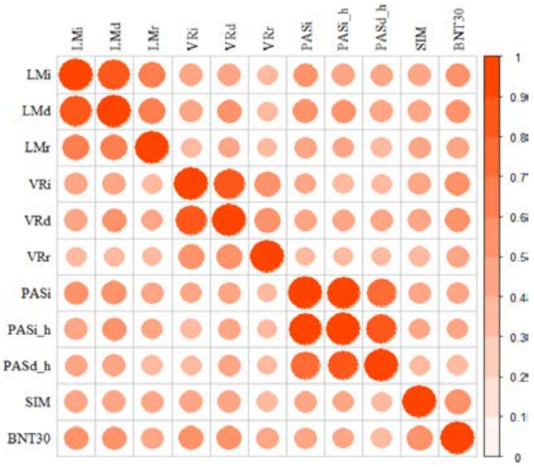

High school and below

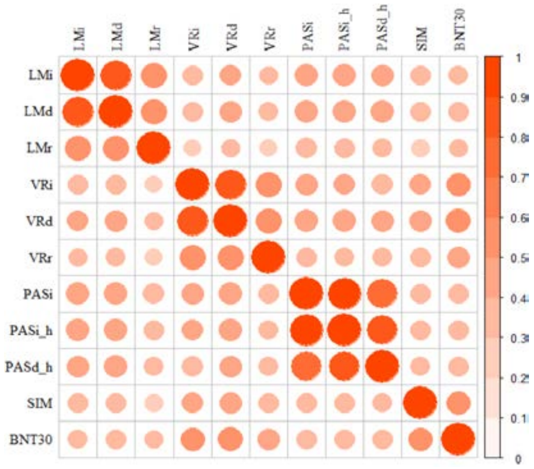

Beyond high school

Supplemental Materials

eFigure 2. The error bars of mean of NP test in three outcome groups. The middle of the bar is the mean. The bar length indicates the standard deviation. Bars are significantly different according to Tukey-Kramer test with Bonferroni correction. The F values for these NP tests are 882.6, 782.3, 567.7, 618.9, 152.4, 408.0, 569.7, 445.5, 432.5, 578.9 and 771.5 respectively. For each F value, *P*<.001. AD: Alzheimer's disease; HC: healthy control; NAD: non-Alzheimer's dementia

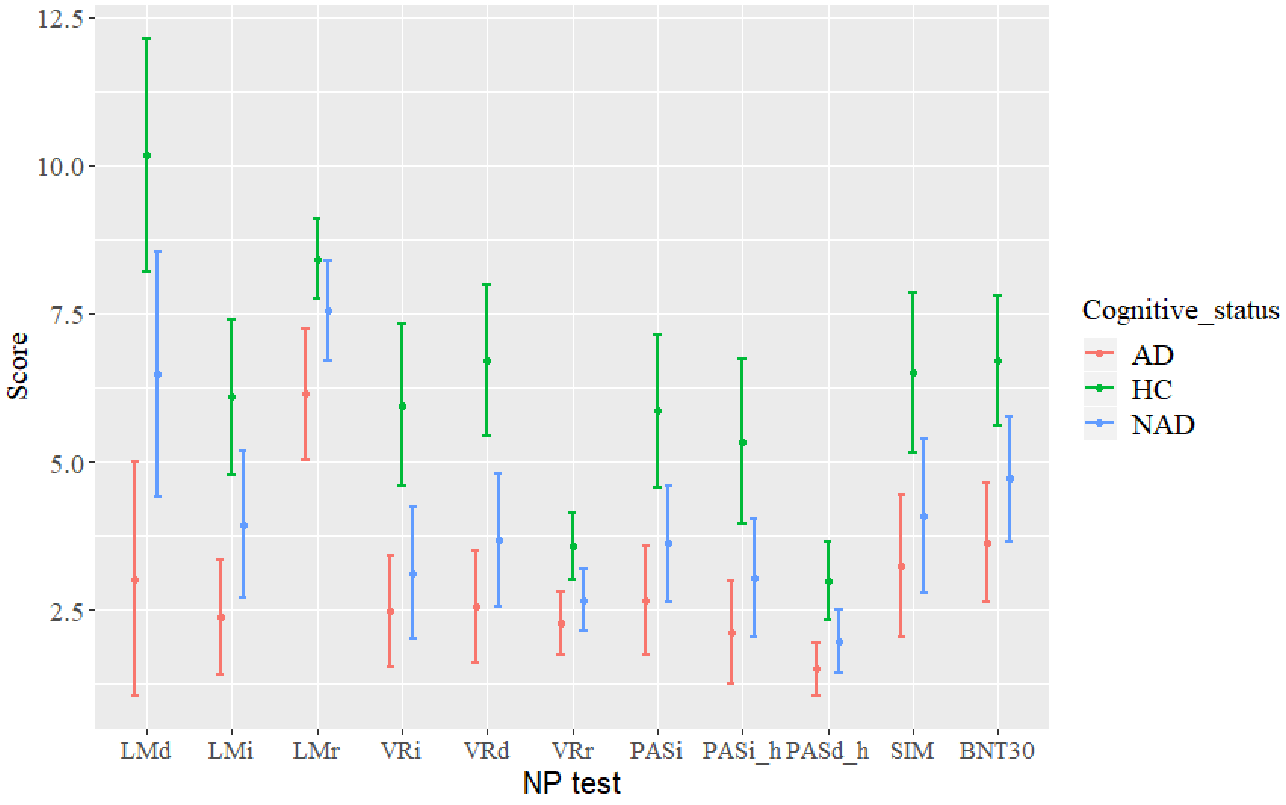

Supplemental Materials

eFigure 3. Hierarchical influence of NP tests and cognitive status in (A) females with ApoE4 alleles; (B) males with ApoE4 alleles. Although sex has an impact on the hierarchical structure of cognitive functions in participants with ApoE4+, the BN structure of females with ApoE4+ remains relatively similar to that of gender-unstratified ApoE4+. In contrast, the BN structure of males with ApoE4+ changed drastically.

A

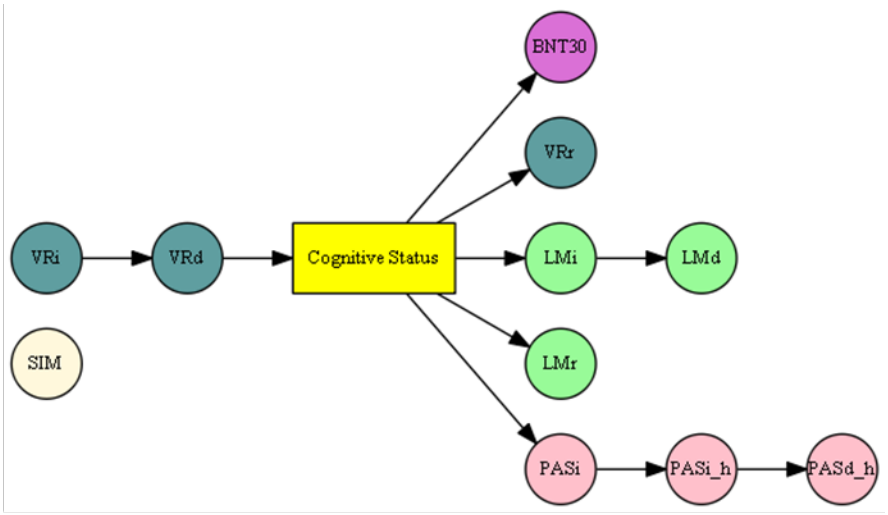

B

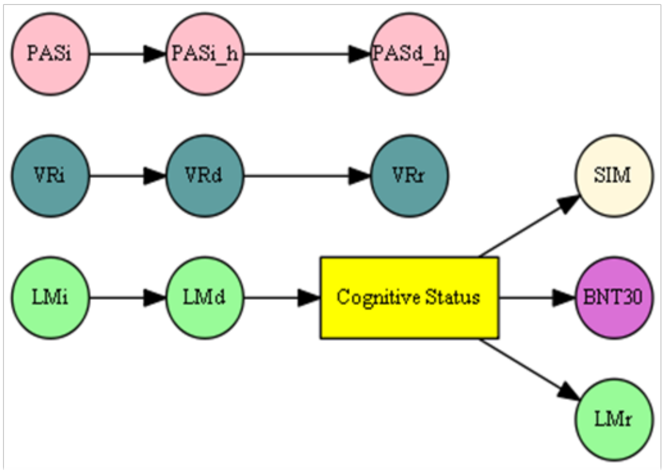

Supplemental Materials

eFigure 4. An example of histogram of probability inference with 100 times experiment.

A) $$P_{mean}(CognitveStatus = HC \mid LMi = 7, LMd = 9) = \frac{\sum_{i=1}^{100} P_i(\text{Cognitive}Status = HC \mid LMi = 7, LMd = 9)}{100} = 0.8899$$

B) $$P_{mean}(CognitveStatus = HC \mid LMi = 7, LMd = 9, VRd = 5) = \frac{\sum_{i=1}^{100} P_i(\text{Cognitive}Status = HC \mid LMi = 7, LMd = 9, VRd = 5)}{100} = 0.9111$$

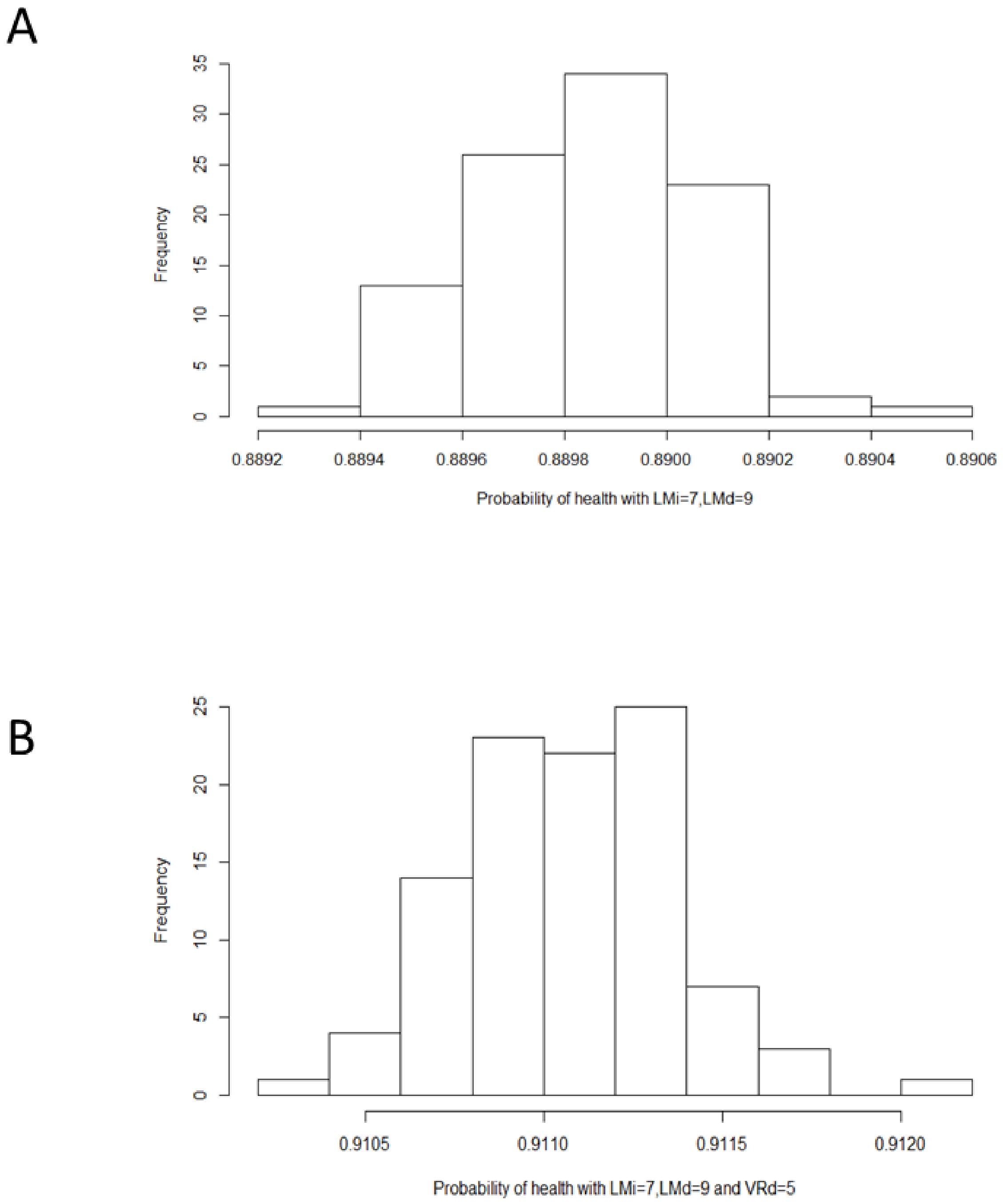

Supplemental Materials

eFigure 5. A heatmap of the Pearson's correlation between NP tests in simulated participants. The red rectangles represent different clusters of tests. Big nodes colored red represent high correlation, while small nodes with white color represent low correlation. NP tests belonging to the same cognitive function tend to cluster together.

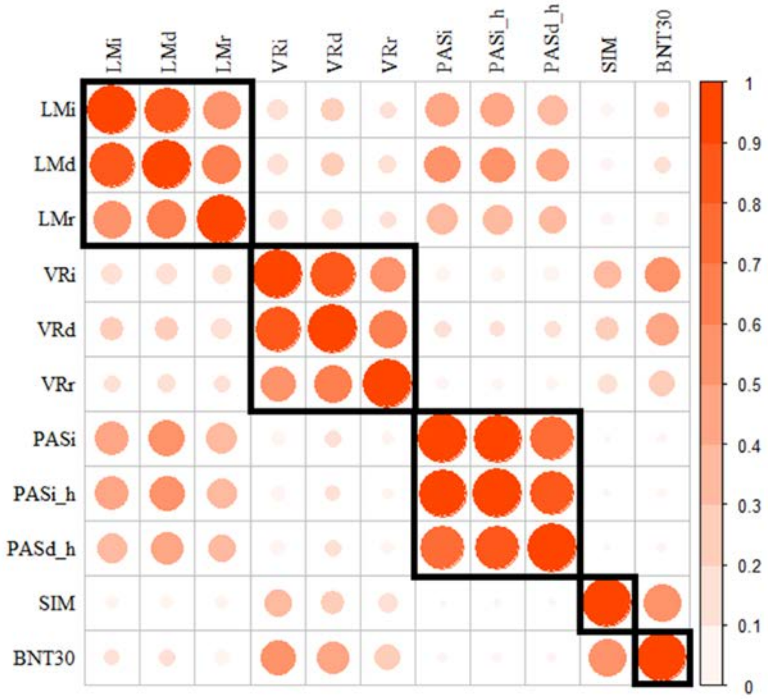

Supplement: Multimedia Appendix 1 [file jmir_v22i4e15376_app1.pdf]
